# Temporal Patterns of Alcohol- and Drug-Related Overdoses During the COVID-19 Pandemic: A National EMS-Based Study by Age and Gender in Israel

**DOI:** 10.3390/ijerph23050619

**Published:** 2026-05-07

**Authors:** Anna Khalemsky, Moshe Z. Abramowitz, Roman Sonkin, Haim Y. Knobler, Eli Jaffe

**Affiliations:** 1 Management Department, Jerusalem Multidisciplinary College, Jerusalem 91010, Israel; 2 Health Management Department, Jerusalem Multidisciplinary College, Jerusalem 91010, Israel; mosheab@edu.jmc.ac.il (M.Z.A.); haim.knobler@gmail.com (H.Y.K.); 3 Hadassah School of Medicine, Hebrew University, Jerusalem 9112102, Israel; 4 Community Division, Magen David Adom, Or Yehuda 6021805, Israel; sonkin@outlook.co.il (R.S.); elijaffe2@gmail.com (E.J.); 5 Faculty of Health Sciences, Ben-Gurion University, Beer-Sheva 8410501, Israel; 6 Department of Emergency Medicine, Ben-Gurion University, Beer-Sheva 8410501, Israel

**Keywords:** COVID-19 pandemic, substance overdose, alcohol-related emergencies, drug-related emergencies, emergency medical services, population surveillance, age differences, gender differences, public health monitoring

## Abstract

**Highlights:**

**Public health relevance—How does this work relate to a public health issue?**

- Examines temporal patterns of alcohol- and drug-related overdose events during the COVID-19 pandemic using nationwide EMS data.
- Provides real-time insights into non-fatal overdose incidents not captured in hospital or mortality records.

**Public health significance—Why is this work of significance to public health?**

- Identifies significant variation in overdose events across distinct pandemic phases and demographic groups.
- Demonstrates the value of EMS data as a complementary surveillance source for acute substance-related harm.

**Public health implications—What are the key implications or messages for practitioners, policy makers and/or researchers in public health?**

- Supports integration of EMS-based indicators into public health surveillance systems for the early detection of emerging risks.
- Informs targeted prevention strategies and resource allocations during periods of societal disruption.

**Abstract:**

The COVID-19 pandemic introduced unprecedented societal disruptions that may have altered patterns of acute substance-related harm. Despite extensive research on overdose mortality, there remains limited real-time, population-level evidence based on Emergency Medical Services (EMS) data. This study provides a national, multi-year characterization of temporal trends in alcohol- and drug-related overdose incidents in Israel across thirteen defined pandemic phases. A total of 18,348 overdose cases recorded between 1 January 2019 and 31 December 2022 were analyzed. Descriptive statistics were calculated, event frequencies were normalized by period length, and loglinear Poisson models were applied to compare occurrences across periods. Cluster analysis was used to explore joint age- and gender-related patterns. Alcohol-related overdoses demonstrated marked fluctuations across pandemic phases, whereas drug-related overdoses showed comparatively moderate variation and a gradual increase over time. Age- and gender-specific heterogeneity was observed across periods. As an observational study based on EMS records, causal inference cannot be established. These findings provide population-level surveillance evidence of dynamic overdose patterns during prolonged societal disruption and highlight the importance of integrating EMS-based monitoring into public health preparedness strategies.

## 1. Introduction

The onset of the COVID-19 pandemic in late 2019 and its rapid global spread triggered profound and prolonged societal disruption. Within months, a highly interconnected world fragmented into localized, confined social environments (Wassler & Talarico, 2021; Paul et al., 2020) [1,2]. For many, the pandemic generated unprecedented challenges to emotional well-being, as mobility constraints, social distancing and prolonged isolation eroded conventional support systems (Paudel, 2021) [3]. Public health crises amplify existing social and behavioral vulnerabilities, including substance use and mental health challenges (Galea et al., 2004) [4]. These impacts are influenced by structural factors such as economic stability and access to healthcare (Abbas, 2021) [5], as well as disruptions to social support, underscoring the need for coordinated system-level responses.

Increases in alcohol- and drug-related overdoses during the pandemic have been linked to multiple interacting factors that vary across populations (Vu et al., 2023) [6]. Heightened stress and economic uncertainty challenged psychological resilience (Godinić & Obrenovic, 2020) [7], while many individuals experienced worsening mental health (Ivbijaro et al., 2020) [8]. Social isolation reduced protective support networks (Clair et al., 2021; Killgore et al., 2020; Szkody et al., 2021) [9,10,11], and increased substance use often served as a coping mechanism despite long-term risks (Singh et al., 2021; Whittaker & Kingston, 2022) [12,13]. Additional contributors include exacerbation of pre-existing psychiatric conditions (Avison & Gotlib, 1994; Verdolini et al., 2021) [14,15] and disruption of daily routines, particularly among certain age groups (Palmer et al., 2023) [16].

Age-related differences shaped how populations experienced the pandemic. Adolescents and young adults faced disruptions in social interactions and routines, increasing susceptibility to substance use (Clendennen et al., 2021; Cousijn et al., 2018; Romm et al., 2022) [17,18,19]. Middle-aged adults experienced sustained stress related to caregiving and economic pressures (Pearman et al., 2021) [20], while older adults were particularly affected by isolation and reduced access to services, elevating risks of substance-related coping (Banerjee et al., 2020) [21].

Gender also influences stress responses and substance-use patterns. Men have traditionally shown higher rates of alcohol and drug use, although this gap has narrowed in younger cohorts (McHugh et al., 2018) [22]. Differences in coping and use motivations have been documented, with women more often using substances for emotional regulation and potentially progressing more rapidly to dependence (Sun & Stewart, 2007; Eschenbeck et al., 2007; Tuchman, 2011; Becker et al., 2017) [23,24,25,26]. These patterns highlight the importance of considering both age and gender in analyzing substance-related emergencies during the pandemic.

Despite substantial international attention to pandemic-related changes in substance use, population-level evidence from real-time Emergency Medical Services (EMS) data remains limited. Much of the existing research relies on mortality records, hospital admissions or self-reported surveys, which do not fully capture non-fatal overdose incidents requiring urgent prehospital interventions. EMS administrative datasets provide a unique surveillance vantage point, offering near-real-time insight into acute, community-level morbidity. In Israel, Magen David Adom (MDA)—the national EMS provider—maintains a unified, nationwide system encompassing all prehospital emergency calls, enabling consistent monitoring across diverse periods of societal disruption. Such data is particularly valuable when healthcare-seeking behaviors, exposures and risk environments change rapidly.

This study analyzes 18,348 alcohol- and drug-related overdose cases attended by Magen David Adom between 1 January 2019 and 31 December 2022. Its objective is to characterize temporal trends across thirteen defined pandemic phases and to examine age- and gender-specific patterns within this national EMS dataset. By leveraging comprehensive real-time surveillance data, this study provides an integrated perspective on demographic variation in acute substance-related emergencies during prolonged societal disruption and offers evidence to inform public health preparedness and response.

## 2. Materials and Methods

### 2.1. Data Source and Study Population

This retrospective observational study used administrative Emergency Medical Services (EMS) records obtained from Magen David Adom (MDA), the national provider of prehospital emergency care in Israel. The dataset included 18,348 ambulance dispatches classified as alcohol- or drug-related overdose between 1 January 2019, and 31 December 2022. Classification of overdose type was based on a combination of standardized EMS dispatch coding and on-scene clinical assessment performed by trained emergency medical personnel. Within the MDA system, dispatch codes categorize incidents as alcohol-related or drug-related overdose based on information obtained during the emergency call. Upon arrival, EMS personnel assess the patient using structured clinical protocols, including level of consciousness, respiratory status, vital signs, and suspected substance exposure based on patient reports, bystander information, or scene characteristics. Alcohol-related overdose cases were defined as incidents involving acute alcohol intoxication requiring medical intervention, while drug-related overdose cases included suspected toxicity from illicit drugs, prescription medications, or mixed substances as identified by EMS personnel. These classifications are recorded in a standardized electronic reporting system. However, they are based on field assessment and available information rather than laboratory confirmation, and some degree of misclassification cannot be excluded.

Variables available for analysis included date and time of dispatch, predefined pandemic period (13 categories), ambulance type, medical dispatch classification (alcohol or drug overdose), patient gender, patient age and clinical indicators recorded at the scene. All records were fully de-identified before analysis. Only cases meeting EMS criteria for alcohol- or drug-related overdose were included; no additional exclusion criteria were applied beyond those described in the data management section.

### 2.2. Definition of Study Periods

The study period spanned four consecutive years, from 1 January 2019 through 31 December 2022. The pre-pandemic baseline was defined as 1 January 2019 to 20 March 2020, corresponding to the period prior to the implementation of COVID-19-related public health measures in Israel. Although COVID-19 emerged globally in late 2019, no restrictions or observable system-level impacts occurred in Israel before March 2020. Therefore, this interval was treated as the pre-pandemic baseline to reflect stable population behavior and EMS utilization patterns within the national context. The remaining 12 periods corresponded to official Ministry of Health definitions of COVID-19 waves and the intervening reopening phases in Israel. Each period reflects documented changes in public health restrictions, mobility policies and national epidemiological conditions. This classification ensured consistency with governmental terminology and avoided data-driven segmentation that could introduce bias. Exact dates for each period are provided in Supplementary Table S1. The predefined periods differ in terms of public health restrictions and epidemiological conditions. Pandemic waves were characterized by increased infection rates and the implementation of stricter measures, including lockdowns, mobility restrictions, and limitations on social gatherings. In contrast, between-wave periods generally involved partial or full reopening, with reduced restrictions and increased population mobility. This classification reflects the broader societal and behavioral context in which EMS utilization occurred and allows for comparison of overdose patterns across distinct phases of the pandemic.

### 2.3. Data Management

To prevent distortion from predictable, event-driven spikes in alcohol-related incidents, specific national holidays associated with atypically high alcohol consumption were excluded: New Year’s Day, Purim, Veterans’ Day and Independence Day. These recurrent events represent culturally established anomalies rather than secular temporal trends. Exclusions were applied uniformly across all study years to maintain comparability between periods and avoid disproportionate influence on any specific pandemic phase. A total of 1148 cases were excluded. These exclusions were applied to reduce confounding from recurring, non-pandemic-related behavioral events. No additional reclassification or validation procedures were applied by the researchers; therefore, all case definitions reflect the original EMS clinical and dispatch classifications.

The dataset contained minimal missing data for the variables included in the analysis. Records with missing values in key variables (age, gender, or dispatch classification) were excluded from the analysis. No imputation procedures were applied.

### 2.4. Statistical Analysis

Statistical analysis proceeded in four stages.

(1) Descriptive analysis. Descriptive statistics were computed for each study period, including the frequency of alcohol- and drug-related overdose incidents, age distributions (continuous and categorical) and gender proportions. Case counts were normalized by period length to enable valid comparison across periods of unequal duration.

(2) Temporal visualization. Normalized case frequencies were plotted across the 13 periods to illustrate overarching temporal trends and differences between alcohol- and drug-related overdoses.

(3) Poisson regression modeling. Log-linear Poisson regression models were fitted with period indicators included as categorical predictors. Model fit was assessed using goodness-of-fit statistics and examination of residuals. The models were specified as parsimonious Poisson regressions with period indicators as the primary explanatory variables, consistent with the descriptive aim of assessing temporal variation. Additional covariates were not included due to data limitations and the focus on unadjusted temporal patterns. Overdispersion was assessed and, when present, robust standard errors were applied to account for variance inflation.

(4) Cluster analysis. Unsupervised k-means clustering was used to examine joint age, gender and dispatch-type patterns within three selected periods (pre-pandemic, first wave and the period between the first and second waves). The number of clusters (k = 4) was selected based on a combination of methodological and practical considerations. The choice was based on interpretability, internal variance reduction and stability across initialization seeds. Given the exploratory aim of the analysis, emphasis was placed on interpretability and meaningful differentiation of demographic patterns (age, gender, and dispatch type). Multiple cluster solutions were examined. Solutions with fewer clusters resulted in overly aggregated profiles that obscured relevant distinctions, whereas solutions with more clusters produced fragmented groupings with limited interpretive value. Stability of the clustering solution was assessed by repeating the analysis across multiple random initializations, yielding consistent cluster structures. Formal optimization criteria (e.g., elbow method or silhouette scores) were not the primary basis for selection, as the goal was descriptive characterization rather than optimization of clustering performance. This approach is consistent with common practice in exploratory clustering analyses in applied health research.

Formal sensitivity analyses were not performed; however, robustness of the findings was supported through consistent analytical approaches, including normalization by period duration and the use of predefined period classifications.

All analyses were conducted using SPSS version 31.0.1.1 (IBM Corp., Armonk, NY, USA) [27]. Cluster analysis was performed using WEKA version 3.8.6 (University of Waikato, New Zealand) [28]. Population denominators were not incorporated; therefore, results represent absolute event counts rather than incidence rates.

## 3. Results

### 3.1. Temporal Distribution of Overdose Cases

**Figure 1:**
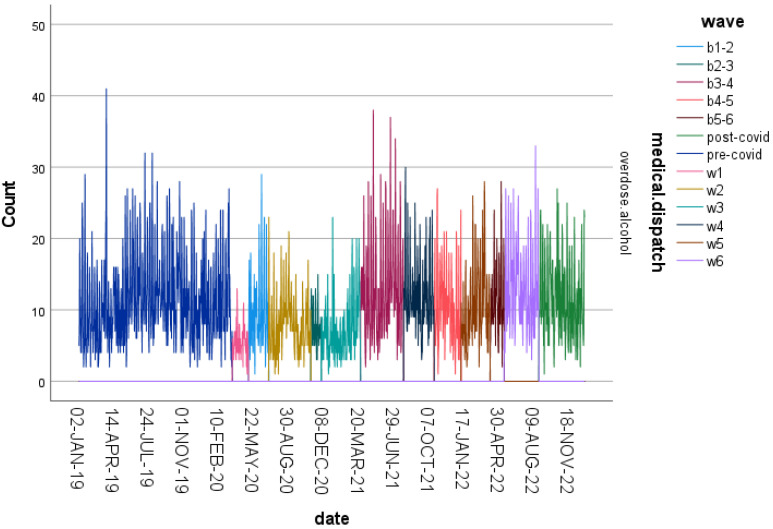
Normalized frequencies of alcohol-related overdose cases across the 13 predefined study periods (2019–2022).

**Figure 2:**
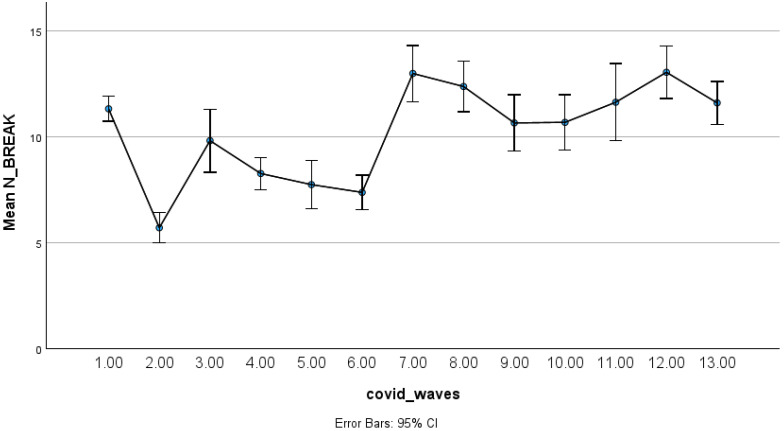
Estimated mean alcohol overdose counts by pandemic phase with 95% confidence intervals.

Alcohol-related overdoses exhibited substantial fluctuations across the 13 study periods, with multiple increases during later pandemic waves compared with the pre-pandemic baseline (Figure 1). These variations suggest sensitivity of alcohol-related emergencies to changes in societal conditions and public health restrictions. To complement the daily time-series visualization, aggregated mean counts with 95% confidence intervals across pandemic phases are presented in Figure 2.

**Figure 3:**
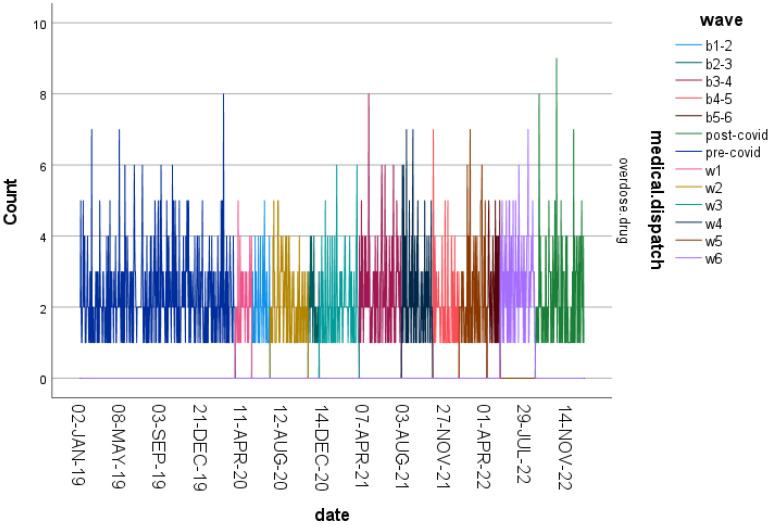
Normalized frequencies of drug-related overdose cases across the 13 predefined study periods (2019–2022).

**Figure 4:**
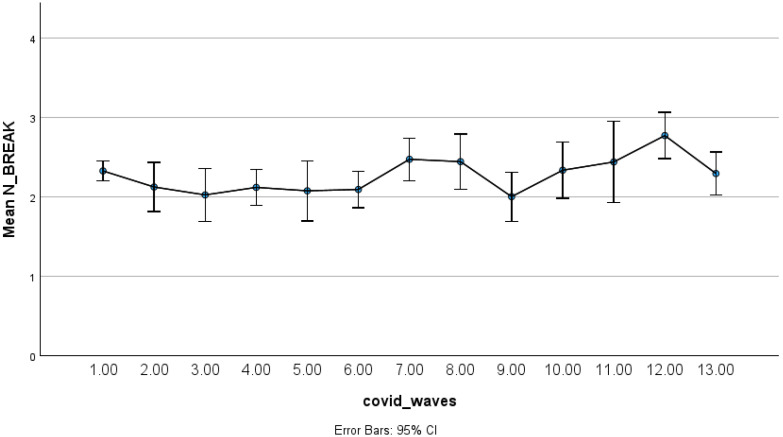
Estimated mean drugs overdose counts by pandemic phase with 95% confidence intervals.

Drug-related overdoses showed a different temporal pattern (Figure 3 and Figure 4), with a modest decline during the earliest pandemic phases followed by a gradual and sustained increase throughout subsequent periods. This pattern indicates longer-term upward trends rather than short-term shifts.

Detailed numerical distributions are provided in Supplementary Table S1.

Loglinear Poisson regression analysis (Supplementary Table S2) indicated statistically significant variation in overdose events across the predefined pandemic phases. Relative to the pre-pandemic baseline, several periods showed significant differences in incidence rates. Alcohol-related overdoses demonstrated greater temporal variability, with multiple phases exhibiting significant increases or decreases compared to baseline. In contrast, drug-related overdoses showed fewer abrupt changes, with patterns suggesting more gradual shifts across later periods. Overall, the regression results confirm significant heterogeneity in overdose occurrence across pandemic phases.

For descriptive purposes, the study periods were also aggregated into three broader categories: pre-pandemic, pandemic, and post-pandemic. This simplified comparison indicates overall differences in overdose patterns across major stages; however, these aggregated results should be interpreted cautiously, as they may mask substantial variation between individual pandemic phases characterized by differing public health measures and societal conditions. When normalized by period duration, alcohol-related overdoses showed similar average daily levels during the pre-pandemic and pandemic periods (~9.8 vs. ~9.7 cases per day), followed by a decline in the post-pandemic phase (~7.5 cases per day). Drug-related overdoses demonstrated a slight increase during the pandemic (~1.9 vs. ~1.8 cases per day pre-pandemic), with lower levels observed post-pandemic (~1.5 cases per day).

### 3.2. Age Distribution Across Study Periods

Across all study periods, individuals aged 21–65 consistently represented the largest proportion of alcohol-related overdose cases, although their relative share varied moderately between phases (Table 1). Detailed age group distributions are provided in Supplementary Table S3. Adolescents and young adults (ages 11–21) showed greater fluctuation, with several increases observed between waves, particularly for alcohol-related incidents.

For drug-related overdoses, age distributions remained comparatively stable over time. Most cases involved individuals aged 21–65, while adolescents accounted for a small and minimally changing proportion across periods.

Mean age values showed limited variability for both overdose types, indicating that pandemic-related shifts were reflected primarily in case frequency rather than demographic restructuring.

### 3.3. Age and Gender Patterns: Cluster Analysis

Cluster analysis identified distinct demographic patterns across the three selected periods (pre-pandemic, first wave and the interval between the first and second waves) (Table 2).

Pre–COVID. Four clusters emerged, reflecting clear demographic differentiation. Two clusters were dominated by men—one consisting primarily of older adults with alcohol-related overdoses and one of younger males with drug-related overdoses. A third cluster represented younger males with alcohol-related overdose, and a fourth cluster comprised predominantly younger females with alcohol-related incidents.

First wave. All clusters showed a high proportion of male cases, though age distributions varied substantially. One cluster consisted mostly of younger individuals, while others reflected progressively older age profiles. Alcohol-related overdoses predominated in most clusters, except one characterized mainly by drug-related incidents.

Between-wave period (B1–2). Cluster configurations shifted relative to earlier phases. Three clusters were composed primarily of men with different age profiles, while one cluster consisted almost exclusively of women. Across clusters, alcohol-related overdoses represent the majority of cases, with drug-related incidents forming a smaller but stable proportion.

These cluster patterns reflect shifts in demographic composition over time. While they do not represent incidence estimates, they reveal meaningful differences in the age- and gender-specific profiles of individuals experiencing overdose during different stages of the pandemic.

## 4. Discussion

This national EMS-based study identified distinct temporal patterns in alcohol- and drug-related overdose incidents across multiple phases of the COVID-19 pandemic in Israel. Alcohol-related overdoses fluctuated more sharply between waves and reopening periods, whereas drug-related overdoses exhibited a steadier long-term increase. These findings align with international reports documenting heterogeneous changes in substance use and related harms during the pandemic, although specific trajectories vary across countries and demographic contexts (Vu et al., 2023; Whittaker & Kingston, 2022) [6,13].

The Poisson regression results support the descriptive findings by confirming that differences in overdose events across pandemic phases are statistically significant. Several phases showed significantly elevated incidence rate ratios, while others demonstrated reduced rates compared to baseline, highlighting the dynamic nature of overdose patterns across the pandemic timeline. The higher variability observed in alcohol-related overdoses may indicate sensitivity to short-term societal changes, whereas the more gradual pattern in drug-related overdoses suggests longer-term underlying processes. These findings should be interpreted as temporal associations rather than causal effects, given the observational design and the absence of population denominators or exposure measures. The consistency between regression results and descriptive analyses strengthens confidence in the robustness of the observed temporal patterns.

The pronounced variability in alcohol-related overdoses coincided with changes in pandemic phases and public health conditions, suggesting sensitivity to broader societal dynamics. Periods with intensified restrictions were temporally associated with lower observed frequencies, whereas reopening phases coincided with increases, although causal relationships cannot be established. The gradual increase in drug-related overdoses was observed across later periods, a pattern that may be consistent with underlying longer-term factors, though these cannot be directly examined in the present study.

Age-specific analyses revealed meaningful heterogeneity across pandemic phases. Adolescents and young adults showed marked fluctuations in involvement with alcohol-related overdoses, consistent with evidence that pandemic-related disruptions disproportionately affected youth routines, social networks and developmental trajectories (Clendennen et al., 2021; Romm et al., 2022) [17,19]. Adults aged 21–65 consistently accounted for most overdose incidents, suggesting that working-age individuals faced ongoing stressors, including employment instability and heightened caregiving burdens (Pearman et al., 2021) [20].

Gender patterns similarly underscored demographic variation. Men represented most overdose cases across all periods, consistent with known gender differences in substance use prevalence and progression (McHugh et al., 2018; Becker et al., 2017) [22,26]. However, the cluster analysis demonstrated that demographic composition was not static: the relative distribution of age and gender groups shifted across phases, particularly during the period between the first and second waves. These findings indicate that prolonged societal disruption may differentially influence subgroups within the population. The differences observed between clusters likely reflect underlying demographic variation in substance-related emergencies rather than discrete causal mechanisms. For example, clusters characterized by predominantly older individuals and a high proportion of alcohol-related overdoses may correspond to patterns of alcohol use more common in older populations, whereas clusters with higher proportions of drug-related overdoses and younger individuals may reflect different substance-use profiles.

However, these interpretations should be considered cautiously. The dataset does not include information on specific substances, socioeconomic characteristics, or behavioral factors, and therefore the cluster patterns cannot be attributed to specific causes. Instead, the findings highlight the presence of distinct demographic profiles associated with different types of overdose events and should be interpreted as descriptive and hypothesis-generating.

A key contribution of this study lies in its use of nationwide EMS administrative data to examine real-time overdose events. Prior research on pandemic-era substance use has relied heavily on mortality records, hospital admissions or self-reported surveys, each of which captures only part of the broader landscape. EMS datasets provide timely indicators of acute morbidity at the community level and can illuminate emerging trends before they appear in hospitalizations or mortality statistics (Handberry et al., 2021; Al Amiry & Maguire, 2021) [29,30]. By analyzing multiple pandemic phases and integrating demographic cluster analysis, this study adds novel population-level evidence on dynamic overdose patterns during an extended public health crisis. Given the observational nature of the data, these findings should be interpreted as descriptive temporal patterns rather than evidence of causal effects.

Several limitations should be noted. First, EMS data captures only incidents resulting in ambulance dispatch and may underestimate the overall burden of overdose events. Second, the dataset lacks geographic, socioeconomic, substance-specific, and clinical outcome variables, limiting assessment of structural determinants, severity, and spatial patterns. Third, changes in healthcare-seeking behavior during the pandemic may have influenced EMS utilization independently of true incidence. Fourth, the observational design precludes causal inference and restricts interpretation to temporal associations.

Case identification relied on EMS dispatch coding and on-scene clinical assessment without toxicological confirmation, introducing potential misclassification, particularly in cases of mixed or unclear substance exposure. In addition, the absence of population denominators or EMS call volume data limits interpretation in terms of incidence rates; thus, findings reflect patterns in EMS-attended events rather than population-level risk.

The exclusion of selected national holidays was intended to reduce recurring, non-pandemic-related variation, but may limit generalizability to the full burden of overdose events. Future studies could model such effects explicitly.

Despite these limitations, the findings provide valuable insight into patterns of acute substance-related emergencies during prolonged societal disruption. The consistency of results across multiple analytical approaches supports their robustness. Integrating EMS data with broader health and socioeconomic information may further enhance public health surveillance and preparedness.

Future research integrating EMS data with hospital records, toxicological data, and population-based registries could provide a more complete understanding of overdose dynamics, including risk factors, substance profiles, and clinical outcomes.

## 5. Conclusions

This national EMS-based study demonstrates that alcohol- and drug-related overdose incidents in Israel changed dynamically across multiple pandemic phases. Alcohol-related overdoses showed marked short-term fluctuations, while drug-related overdoses followed a steadier, long-term upward trajectory. Meaningful differences were also observed across age and gender groups, emphasizing the importance of population-sensitive approaches to monitoring substance-related emergencies.

The findings highlight the unique value of EMS administrative data as a near-real-time surveillance resource capable of detecting rapid changes in acute substance-related harm during periods of societal disruption. Incorporating EMS indicators into routine public health monitoring frameworks may enhance early detection capacity, improve situational awareness and support more targeted and adaptable intervention strategies during future public health emergencies. Continued integration of EMS data with broader health and socioeconomic information is warranted to better understand the drivers of temporal shifts in overdose patterns and to strengthen evidence-based public health preparedness and response.

From a public health perspective, the findings underscore the potential value of EMS data as a real-time surveillance tool for monitoring acute substance-related harm. Temporal and demographic patterns identified in this study may assist policymakers in identifying high-risk periods and population groups, enabling more targeted prevention and response strategies.

Integrating EMS-based indicators into national surveillance frameworks could enhance early warning capabilities, support resource allocation, and improve coordination between prehospital services and public health systems during future public health emergencies. Such integration may be particularly valuable in contexts where hospital or mortality data are delayed or incomplete.

## Figures and Tables

**Table 1:** Descriptive characteristics of alcohol- and drug-related overdose cases across study periods.

Period	Alcohol N	Mean Age (SD)	% Male	Drug N	Mean Age (SD)	% Male
Pre–COVID	4372	31.55 (15.02)	67.9%	816	33.76 (13.17)	74.5%
W1	237	39.11 (15.33)	75.2%	78	36.02 (14.59)	75.9%
B1–2	511	33.10 (15.91)	70.5%	82	36.71 (13.92)	75.2%
W2	881	34.67 (15.57)	70.4%	176	34.96 (14.27)	68.8%
B2–3	211	34.49 (15.97)	74.1%	52	35.72 (12.84)	69.6%
W3	746	34.50 (16.09)	68.4%	197	35.93 (14.00)	63.8%
B3–4	1464	31.06 (14.84)	65.5%	236	35.53 (13.75)	72.8%
W4	969	30.95 (14.47)	64.7%	173	33.46 (13.93)	68.6%
B4–5	738	31.87 (14.80)	67.8%	119	34.49 (14.03)	63.1%
W5	801	33.72 (15.71)	65.8%	156	33.95 (13.69)	68.9%
B5–6	412	33.00 (14.87)	64.9%	71	34.08 (16.25)	62.8%
W6	1155	33.00 (15.40)	67.7%	223	35.34 (14.56)	61.4%
Post–COVID	1358	33.33 (15.23)	64.4%	262	34.37 (15.20)	62.5%

**Table 2:** Cluster analysis results for Pre-COVID, 1st wave and B1-2 study periods.

	Cluster 1	Cluster 2	Cluster 3	Cluster 4
Pre–COVID 1 January 12019–20 March 2020				
N cases	1409 (24.1%)	644 (11%)	2157 (36.9%)	1635 (28%)
Gender				
Male	1372 (97.4%)	531 (82.5%)	2125 (98.5%)	0 (0%)
Female	11 (0.8%)	105 (16.3%)	0 (0%)	1611 (98.5%)
Other	26 (1.8%)	8 (1.2%)	32 (1.5%)	24 (1.5%)
Age	46.55	34.12	25.20	27.25
Medical dispatch				
Alcohol overdose	1333 (94.6%)	59 (9.2%)	2040 (94.6%)	1524 (93.2%)
Drug overdose	76 (5.4%)	585 (90.8%)	117 (5.4%)	111 (6.8%)
Wave 1 21 March 2020–5 May 2020				
N cases	120 (34.4%)	64 (18.3%)	15 (4.3%)	150 (43%)
Gender				
Male	85 (70.8%)	48 (75%)	11 (73.3%)	119 (79.3%)
Female	35 (29.2%)	16 (25%)	4 (26.7%)	31 (20.7%)
Age	24.46	37.26	44	49.35
Medical dispatch				
Alcohol overdose	102 (85%)	2 (3.1%)	15 (100%)	143 (95.3%)
Drug overdose	18 (15%)	62 (96.9%)	0 (0%)	7 (4.7%)
B1–2 6 May 2020–2 July 2020				
N cases	175 (26.5%)	179 (27.1%)	189 (28.6%)	117 (17.7%)
Gender				
Male	174 (99.4%)	177 (98.9%)	0 (0%)	116 (99.1%)
Female	0 (0%)	0 (0%)	187 (98.9%)	0 (0%)
Other	1 (0.6%)	2 (1.1%)	2 (1.1%)	1 (0.9%)
Age	20.86	36.38	28.3	56.92
Medical dispatch				
Alcohol overdose	156 (89.1%)	149 (83.2%)	166 (87.8%)	98 (83.8%)
Drug overdose	19 (10.9%)	30 (16.8%)	23 (12.2%)	19 (16.2%)

## Data Availability

The EMS dataset analyzed in this study is held by Magen David Adom (MDA). Due to privacy and regulatory restrictions, the data cannot be publicly shared. Access may be granted upon reasonable request and approval from MDA.

## Supplementary Materials

The following supporting information can be downloaded at: https://www.mdpi.com/article/10.3390/ijerph23050619/s1, Table S1: Number of alcohol and drugs overdose cases between 1 January 2019, and 31 December 2022 according to 14 COVID-19 waves and between-waves periods. Table S2: Loglinear Poisson model for comparison of number of occurrences. Table S3: Alcohol and Drug cases—age distribution.
