# Supplementary material for: Temporal Patterns of Alcohol- and Drug-Related Overdoses During the COVID-19 Pandemic: A National EMS-Based Study by Age and Gender in Israel
Source: Int J Environ Res Public Health. 2026 May 7;23(5):619. doi: 10.3390/ijerph23050619 (PMC13207172; doi:10.3390/ijerph23050619)
Supplement: Supplementary file 1 [file ijerph-23-00619-s001.zip › ijerph-4199798-supplementary.pdf]

Supplementary material

Table S1: Number of alcohol and drug overdose cases between Jan. 1, 2019, and Dec. 31. 2022 according to 13 Covid-19 waves and between-waves periods

Time period	N. days	Cases alcohol overdose	**Norm. cases alcohol overdose**	Cases drugs overdose	**Norm. cases drugs overdose**	Total	**Norm. total**
**Pre-Covid**<br>*Jan 1, 2019-March 20, 2020*	438	4956	**11.32**	889	**2.03**	5845	**13.34**
**Wave 1**<br>*March 3, 2020-May 5, 2020.*	45	262	**5.82**	87	**1.93**	349	**7.76**
**B1-2**<br>*May 6, 2020-July 2, 2020*	57	569	**9.98**	91	**1.6**	660	**11.58**
**Wave 2**<br>*July 3, 2020-Oct. 30, 2020*	120	991	**8.26**	199	**1.66**	1190	**9.92**
**B2-3**<br>*Oct. 31, 2020-Nov. 29, 2020*	30	232	**7.73**	56	**1.87**	288	**9.6**
**Wave 3**<br>*Nov. 30, 2020-March 23, 2021*	112	825	**7.37**	207	**1.85**	1032	**9.21**
**B3-4**<br>*March 24, 2021-July 25, 2021*	123	1597	**12.98**	257	**2.09**	1854	**15.07**
**Wave 4**<br>*July 26, 2021-Oct. 20, 2021*	87	1076	**12.37**	188	**2.16**	1264	**14.53**
**B4-5**<br>*Oct. 21, 2021-Jan. 7, 2022*	77	820	**10.65**	130	**1.69**	950	**12.34**
**Wave 5**<br>*Jan. 8, 2022-Apr. 3, 2022*	84	886	**10.55**	161	**1.92**	1047	**12.46**
**B5-6**<br>*Apr. 4, 2022-May 14, 2022*	40	465	**11.63**	78	**1.95**	543	**12.58**
**Wave 6**<br>*May 15, 2022-Aug. 21, 2022*	97	1291	**13.31**	241	**2.48**	1532	**15.79**
**Post-Covid**<br>*Aug. 22, 2022-Dec. 31, 2022*	131	1519	**11.6**	275	**2.1**	1794	**13.69**
**Total**		**15489**		**2859**		**18348**	

Table S2: Loglinear Poisson model for comparison of number of occurrences

	Pre-covid	W1	B1-2	W2	B2-3	W3	B3-4	W4	B4-5	W5	B5-6	W6	Post-covid
Pre-covid	---	-0.67<br><0.001	-0.13<br>0.004	-0.32<br><0.001	-0.38<br><0.001	-0.43<br><0.001	0.14<br><0.001	0.09<br>0.03	-0.06<br>0.15	-0.07<br>0.10	0.03<br>0.52	0.16<br><0.001	0.02<br>0.56
W1	-0.05<br>0.62	---	0.54<br><0.001	0.35<br><0.001	0.28<br><0.001	0.24<br><0.001	0.80<br><0.001	0.75<br><0.001	0.60<br><0.001	0.60<br><0.001	0.69<br><0.001	0.83<br><0.001	0.69<br><0.001
B1-2	-0.24<br>0.02	-0.19<br>0.08	---	-0.19<br><0.001	-0.26<br><0.001	-0.30<br><0.001	0.26<br><0.001	0.22<br><0.001	0.07<br>0.14	0.06<br>0.21	0.15<br><0.001	0.29<br><0.001	0.15<br><0.001
W2	-0.2<br>0.054	-0.15<br>0.16	0.04<br>0.74	---	-0.07<br>0.19	-0.11<br>0.02	0.45<br><0.001	0.40<br><0.001	0.25<br><0.001	0.25<br><0.001	0.34<br><0.001	0.48<br><0.001	0.34<br><0.001
B2-3	-0.08<br>0.42	-0.03<br>0.76	0.16<br>0.15	0.12<br>0.26	---	-0.05<br>0.35	0.52<br><0.001	0.47<br><0.001	0.32<br><0.001	0.31<br><0.001	0.41<br><0.001	0.54<br><0.001	0.41<br><0.001
W3	-0.09<br>0.36	-0.04<br>0.68	0.15<br>0.18	0.11<br>0.31	-0.01<br>0.92	---	0.57<br><0.001	0.52<br><0.001	0.37<br><0.001	0.36<br><0.001	0.46<br><0.001	0.59<br><0.001	0.45<br><0.001

B3-4	0.03<br>0.77	0.08<br>0.43	0.27<br>0.01	0.23<br>0.03	0.11<br>0.27	0.12<br>0.23	---	-0.05<br>0.23	-0.20<br><0.001	-0.21<br><0.001	-0.11<br>0.007	0.03<br>0.52	-0.11<br>0.005
W4	0.06<br>0.53	0.11<br>0.26	0.3<br>0.004	0.26<br>0.01	0.14<br>0.15	0.16<br>0.12	0.03<br>0.73	---	-0.15<br><0.001	-0.16<br><0.001	-0.06<br>0.13	0.07<br>0.06	-0.06<br>0.12
B4-5	-0.18<br>0.08	-0.13<br>0.21	0.06<br>0.62	0.02<br>0.87	-0.1<br>0.34	-0.09<br>0.40	-0.21<br>0.04	-0.25<br>0.02	---	-0.01<br>0.83	0.09<br>0.04	0.22<br><0.001	0.09<br>0.04
W5	-0.06<br>0.58	-0.005<br>0.96	0.18<br>0.09	0.15<br>0.17	0.03<br>0.80	0.04<br>0.72	-0.09<br>0.40	-0.12<br>0.24	0.13<br>0.23	---	0.1<br>0.02	0.23<br><0.001	0.10<br>0.03
B5-6	-0.04<br>0.67	0.01<br>0.92	0.20<br>0.06	0.16<br>0.13	0.04<br>0.68	0.05<br>0.61	-0.07<br>0.49	-0.10<br>0.30	0.14<br>0.17	0.02<br>0.88	---	0.14<br><0.001	-0.003<br>0.95
W6	0.2<br>0.03	0.25<br>0.01	0.44<br><0.001	0.4<br><0.001	0.28<br>0.004	0.29<br>0.003	0.17<br>0.07	0.14<br>0.14	0.38<br><0.001	0.26<br>0.008	0.24<br>0.01	---	-0.14<br><0.001
Post-covid	0.03<br>0.73	0.08<br>0.40	0.27<br>0.01	0.24<br>0.02	0.12<br>0.25	0.13<br>0.21	0.005<br>0.96	-0.03<br>0.77	0.22<br>0.04	0.09<br>0.37	0.07<br>0.46	-0.17<br>0.08	---

Table S3: Alcohol and Drug cases – age distribution

	Pre-covid	W1	B1-2	W2	B2-3	W3	B3-4	W4	B4-5	W5	B5-6	W6	Post-covid
**Alcohol overdose**													
Age groups:<br>*11-15*<br>*15-18*<br>*18-21*<br>*21-65*<br>*65+*	2.3%<br>13.1%<br>16%<br>66.3%<br>2.3%	1.9%<br>5.3%<br>8.4%<br>79.8%<br>4.6%	2.6%<br>13.5%<br>11.2%<br>69.2%<br>3.3%	3.7%<br>10.1%<br>11.0%<br>72.1%<br>3.0%	4.7%<br>11.4%<br>14.2%<br>66.8%<br>2.8%	4.2%<br>11.3%<br>13.4%<br>68.0%<br>3.2%	2.7%<br>13.7%<br>17.6%<br>62.6%<br>3.4%	1.9%<br>11.5%<br>19.9%<br>64.3%<br>2.5%	2.6%<br>14.0%<br>14.5%<br>65.4%<br>3.5%	2.4%<br>11.5%<br>17.2%<br>66.8%<br>2.1%	3.2%<br>10.7%<br>12.1%<br>71.4%<br>2.7%	2.5%<br>10.7%<br>17.1%<br>65.7%<br>4.0%	2.4%<br>8.8%<br>16.9%<br>68.3%<br>3.6%
**Drugs overdose**													
Age groups:<br>*11-15*<br>*15-18*<br>*18-21*<br>*21-65*<br>*65+*	1.0%<br>4.9%<br>9.2%<br>82.6%<br>2.2%	1.1%<br>2.3%<br>9.2%<br>83.9%<br>3.4%	0.0%<br>2.2%<br>9.9%<br>85.7%<br>2.2%	1.0%<br>2.5%<br>10.1%<br>82.9%<br>3.5%	1.8%<br>1.8%<br>5.4%<br>89.3%<br>1.8%	1.4%<br>4.3%<br>5.3%<br>86.0%<br>2.9%	1.2%<br>3.1%<br>7.8%<br>84.8%<br>3.1%	1.2%<br>5.2%<br>11.0%<br>79.2%<br>3.5%	0.0%<br>4.2%<br>9.2%<br>84.0%<br>2.5%	0.6%<br>3.8%<br>13.5%<br>77.6%<br>4.5%	0.0%<br>5.6%<br>18.3%<br>70.4%<br>5.6%	1.3%<br>4.0%<br>10.3%<br>81.2%<br>3.3%	0.0%<br>5.6%<br>18.3%<br>70.4%<br>5.6%
